# Using Ethereum Smart Contracts to Store and Share COVID-19 Patient Data

**DOI:** 10.7759/cureus.21378

**Published:** 2022-01-18

**Authors:** Sai Batchu, Karan Patel, Owen S Henry, Aleem Mohamed, Ank A Agarwal, Henna Hundal, Aditya Joshi, Sankeerth Thoota, Urvish K Patel

**Affiliations:** 1 Neurosciences, Independent Researcher, Montville, USA; 2 Medical Student, Cooper Medical School of Rowan University, Camden, USA; 3 Medical Student, Stanford University School of Medicine, Stanford, USA; 4 Medical Education, Johns Hopkins University School of Medicine, Baltimore, USA; 5 Orthopaedics, Cooper Medical School of Rowan University, Camden, USA; 6 Medicine, Meenakshi Medical College and Research Institute, Enathur, IND; 7 Public Health and Neurology, Icahn School of Medicine at Mount Sinai, New York, USA

**Keywords:** health security, block chain, patient data, ethereum smart contracts, covid

## Abstract

Introduction

The emergence and rapid spread of the coronavirus disease 2019 (COVID-19) pandemic have revealed the limitations in current healthcare systems to handle patient records securely and transparently, and novel protocols are required to address these shortcomings. An attractive option is the use of Ethereum smart contracts to secure the storage of medical records and concomitant data logs. Ethereum is an open-source platform that can be used to construct smart contracts, which are collections of code that allow transactions under certain parameters and are self-executable.

Methods

The present study developed a proof-of-concept smart contract that stores COVID-19 patient data such as the patient identifier (ID), variant, chest CT grade, and significant comorbidities. A sample, fictitious patient data for the purpose of testing was configured to a private network. A smart contract was created in the Ethereum state and tested by measuring the time to insert and query patient data.

Results

Testing with a private, Proof of Authority (PoA) network required only 191 milliseconds and 890 MB of memory per insertion to insert 50 records while inserting 350 records required 674 milliseconds and similar memory per insertion, as memory per insertion was nearly constant with the increasing number of records inserted. Retrieving required 912 MB for a query involving all three fields and no wildcards in a 350-record database. Only 883 MB was needed to procure a similar observation from a 50-record database.

Conclusion

This study exemplifies the use of smart contracts for efficient retrieval/insertion of COVID-19 patient data and provides a case use of secure and efficient data logging for sensitive COVID-19 data.

## Introduction

The ongoing coronavirus disease 2019 (COVID-19) pandemic has revealed the limitations in current healthcare systems to handle patient records. Specifically, the reliance on several entities to communicate sensitive patient information has raised questions related to security [1-3] and transparency [4-7], which has become even more pronounced during the pandemic [8-9]. Medical records of patients contain private information, such as past medical history, significant comorbidities, and lung CT scan grading. Access to such data not only allows for efficient medical treatment but also for health officials to track the progress of COVID-19. However, these abilities depend on the security of such records. Furthermore, as data of such nature are often held in databases using multi-level permission security, access and upload of time-sensitive information are affected. Blockchain technology is gaining traction in healthcare applications and has the potential to mitigate these issues.

Ethereum is a unique type of blockchain as it allows blockchain technology to adapt to software applications. For example, one could construct programs that run if and only certain requirements and conditions are satisfied. These programs are referred to as smart contracts, which are self-executable pieces of code that remain within the Ethereum state and allow certain transactions when called for [10]. Ethereum platform allows such code to be run by providing certain programmable logic requirements. The platform also allows for data storage not limited to cryptocurrency. These characteristics allow for the platform and its smart contracts to be molded to other use cases that require robust integrity and amenability, such as holding patient data. For example, Ethereum blockchain has been shown to handle pharmacogenomics data comprising gene-variant-drug combinations, as well as neuroimaging data [11-12]. No studies, however, have so far investigated the implementation of Ethereum blockchain using COVID-19 patient data. The present study describes a case use of a smart contract to incorporate COVID-19 patient data into the Ethereum platform to enhance patient privacy, records security, and data immutability.

## Materials and methods

We present here a quick, efficient, and memory-saving smart contract to store and query COVID-19-related patient data in Ethereum. A brief overview of Ethereum is provided here. The Ethereum yellow paper contains additional technical explanations [13].

Explanation of smart contracts and the Ethereum system

**Figure 1:**
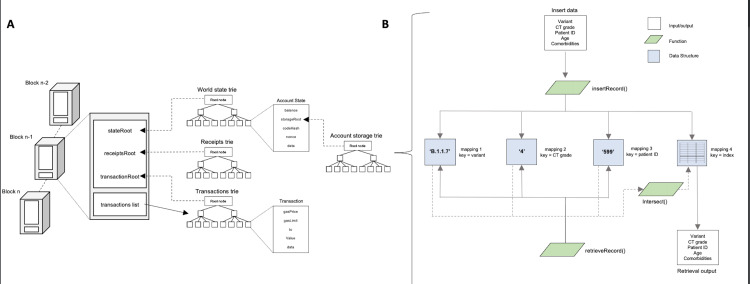
(A) Overview of Ethereum blockchain and (B) proof-of-concept smart contract architecture

The major data structure that Ethereum uses is modified Merkle Patricia tries. Instead of needing to store all the data in the blockchain, these tries allow the blocks to only store the root node hash of each trie, all the while preserving immutability (Figure 1A). The major trie types in Ethereum include account storage trie, world state trie, transaction receipt trie, and transaction trie [13]. The world state is analogous to the global state that is frequently updated by executing transactions and maps account states and addresses (accounts). Information associated with certain accounts and smart contract data is in the account storage trie.

Smart contracts can be described as self-executable, Turing-complete: programs that perform within the Ethereum Virtual Machine (EVM) and maintain their own unique storage [13]. Operations within these contracts are allowed three space types for storing data. These types include memory, stack, and long-term storage. The contract’s storage continues to exist after the computation has finished, as opposed to stack and memory [10]. Ethereum’s native cryptocurrency, Ether, is used to pay costs; colloquially termed ‘gas’, these costs are associated with calling, storing, and retrieving smart contracts [10]. Smart contracts are programmed using an object-oriented programming language designed specifically for EVM, called Solidity [14].

Network nodes must agree as to which transactions are allowed to be added to the blockchain through consensus mechanisms. One such mechanism, Proof of Work (PoW) consensus, requires “mining” of blocks by nodes to solve a mathematical puzzle utilizing computational energy. Only when the puzzle is solved and validated by other nodes on the network is the block aggregated to the chain, and a reward is given to the node that succeeded in mining the block. A faster alternative to PoW is the Proof of Authority (PoA) consensus. This mechanism consists of certain trusted nodes, known as ‘authorities’, which are the only nodes allowed to add new blocks after validating transactions. Since this type of mechanism is faster than PoW, it is commonly utilized in configuring private blockchain networks [15].

Network configuration

Querying and storing sample patient data were experimented via smart contracts in Truffle suite v5.1.62, an Ethereum development framework equipped with a JavaScript testing environment. The development network was private and separate from the public Ethereum network, which allowed for testing without the need for constantly deploying. The contracts were run using the PoA consensus with six nodes on the network. As node configurations do not change performance in private PoA networks, different configurations were not assessed [16]. Default gas limits were utilized throughout smart contract testing. The smart contract code is available at https://github.com/Batchu-Sai/covid19.

Database architecture and insertion

Using arrays to store data requires iteration for observation retrieval. To circumvent this, mappings were implemented, which are conducive to efficient key-value lookups. Four storage mappings were implemented with Solidity’s mapping type. Every observation was placed in its individual struct, which is a special type to group numerous variables of heterogeneous types. Fictitious patient data was used for one mapping. The other three mappings - the variant, comorbidities, and patient identifier (ID) - acted as keys to an array of counter IDs. This counter ID variable assigns a custom index for each observation insertion and is globally updated. This architecture allows using any of the three keys to retrieve the inserted patient observation using the specific counter IDs (Figure 1B) and likewise allows for non-iterative lookup for more efficient database querying.

**Figure 2:**
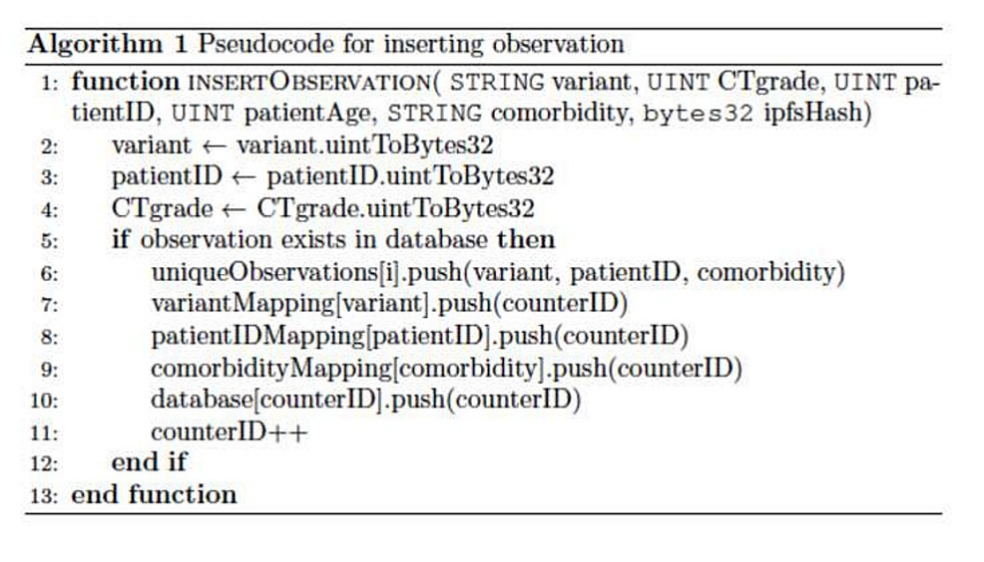
Pseudocode for inserting observation

A function was written to insert a record for a single patient for a specific variant-patient ID-comorbidity combination (Figure 2). Fictitious patient data was constructed, which included the COVID-19 variant, chest CT severity grade, patient ID, patient age, and significant comorbidities. If required, the function first converts the patient data to the desired storage type. Then the function checks if the combination already exists. If not, the variant-patient ID-comorbidity combination is aggregated to an array. The variant, patient ID, and comorbidities are utilized to key into their mappings. The counter ID is appended to the value array. The counter ID variable is updated after the observation struct is inserted into the database with the key-value pair (comprised of the counter ID - patient data struct).

The variant, CT scan grade, patient ID, and age were stored as bytes32 variables. The comorbidities field was treated as a string since comorbidities may be long descriptions and patients uncommonly present with several comorbidities.

Querying database

**Figure 3:**
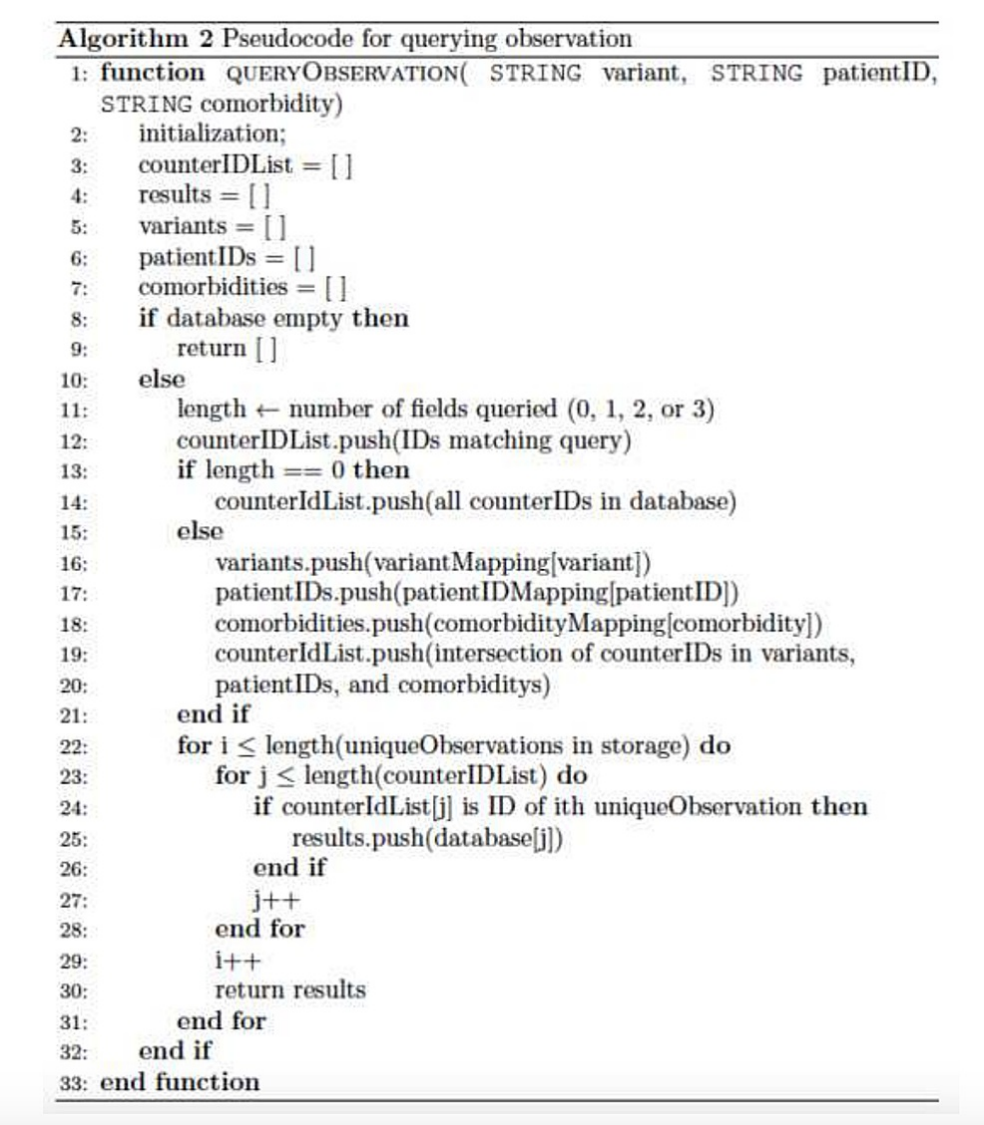
Pseudocode for querying observation

A function used to query the observations was written to take up to three fields (variant, patient ID, and comorbidities) along with wildcards to query by (Figure 3). Initially, the function examines which fields have been queried. These fields are subsequently utilized as keys to retrieve the corresponding values from the counter ID mapping. If wildcards were used to query, then all the counter IDs are returned. For each record, the minimum length counter ID array is looped through to examine if it equals the counter ID from the outer loop. The matching ID is taken to collect the value of the struct from the corresponding database mapping. The framework was adapted from Gürsoy et al. [11]. 

## Results

**Figure 4:**
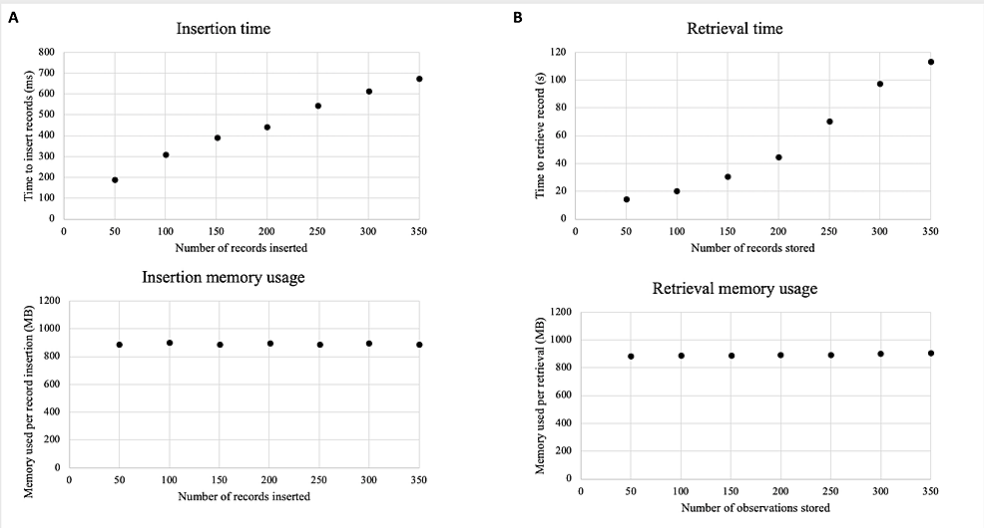
(A) Insertion time and memory usage. (B) Retrieval time and memory usage

JavaScript console with external scripts tested the smart contract. To confirm accuracy, 50 stochastic queries were manually checked with the fields used to query and were confirmed to retrieve accurate results in every case. Time and memory for inserting and querying were also measured, with varying numbers of records stored in the database (Figure 4). The time to insert 50 records was 191 milliseconds while inserting 350 records required 674 milliseconds. Inserting 50 records required 890 MB of memory per insertion. To insert 350 records required nearly the equivalent memory per insertion (Figure 4A). Retrieving required nearly 912 MB for a query involving all three fields and no wildcards in a 350-record database. Only 883 MB was needed to procure a similar observation from a 50-record database (Figure 4B). Retrieving from a 50-record database with similar query characteristics required 16 seconds while a larger 350-record database showed a 112-second retrieval time.

## Discussion

In this study, we presented a proof-of-concept and use case smart contract programmed in Solidity using the Ethereum blockchain. This smart contract demonstrated the ability to store and retrieve COVID-19 patient data. The method’s speed and memory per insertion were measured with varying database record sizes, demonstrating that the technique is both rapid and readily feasible. This demonstrated a practical method while also allowing for efficient memory and time usage when storing and retrieving observations. The results show that blockchain technology can be used to solve problems not specific to cryptocurrency. The results also exemplify the practicality and efficiency of smart contracts involving health-related data.

Secure maintenance of COVID-19 patient medical data is critical, as data corruption can lead to fatal medical errors and misleading public health statistics. Moreover, data should only be accessible to authorized persons such as healthcare workers and researchers. Solutions utilizing blockchain technology, such as smart contracts, could prevent centralized point-of-failure scenarios, create an immutable database, and allow decentralization, ultimately mitigating the probability of data corruption. Indeed, blockchain technology is being conceptualized in other fields as well [11-12,17-20].

During the COVID-19 pandemic, patients have presented with a wide array of symptoms. As part of diagnosing the condition, distinct criteria needed to be defined and met. Lung and infectious disease experts have noted that an especially unique factor of COVID-19 patients is the sound of their breathing, speaking, and dry coughing [21-24]. To diagnose patients’ conditions and create tools that can diagnose them in physician-lacking settings, scientists have created machine-learning algorithms. These tools require large datasets as input, and their final product algorithms can rapidly and automatically function in clinics. In this case, algorithms were created to diagnose COVID-19 based on these sounds. Similarly, associations have been found between other symptoms and COVID-19. The creation of such tools is highly dependent on large datasets and their secure handling. In pandemics and other situations with large datasets containing private patient information, the Ethereum blockchain can be used to speed up secure data storage and retrieval, facilitating the faster and safer development of clinical tools.

As with any novel development, the Ethereum platform consists of limitations associated with smart contracts. For instance, programming and deploying smart contracts require knowledge of the Solidity language, which comprises numerous idiosyncrasies. Legal ramifications also need to be considered when dealing with smart contracts, since they reduce dependence on intermediaries, including lawyers. Therefore, it is imperative that all parties working with smart contracts understand the legal nomenclature of smart contract law. There is also the possibility of delayed transactions in instances where blockchain congestion results in delayed transactions and increase transaction costs. Such limitations exemplify the demand for a stabler platform. Future studies should focus on how such smart contract methods can also be applied to other problems in the medical research community. Additionally, future studies should also focus on other smart contract architectures best suited for other types of medical data. However, the potential of using smart contracts for COVID-19 information remains.

## Conclusions

Blockchain technology can be used to store and retrieve COVID-19-related information securely, immutably, and rapidly. The Ethereum blockchain presents several advantages and has not yet been applied to healthcare information storage, especially during pandemics. In this study, we presented a method for securely and rapidly storing and retrieving COVID-19-related health information using smart contracts with the Ethereum blockchain. It enables the protection of patient information while facilitating more efficient research and communication.
